# Ankylosis and Its Treatment

**Published:** 1899-07-01

**Authors:** A. H. Tubby

**Affiliations:** Senior Assistant Surgeon to Westminster Hospital, Surgeon to the National Orthopædic and Evelina Hospitals


### Hospital Clinics and Medical Progress.

### ANKYLOSIS AND ITS TREATMENT.

By A. H. Tubby, M.S.Lond., F.R.C.S.Eng., Senior Assistant Surgeon to Westminster Hospital, Surgeon to the National Orthopædic and Evelina Hospitals.

(*Continued from page* 210.)

In giving an opinion it is important to notice whether the contraction and malposition be extra-articular or intra-articular. The range of movement in the affected joint should be compared with that in the corresponding and sound joint, usually under an anæsthetic, but no force should be used. In forming an idea as to the possibility of obtaining an increase of movement, the cause of the deformity should be carefully inquired into. Ankylosis after tuberculous disease is generally best left alone, unless the disease has subsided for some years.

The position in which ankylosis has occurred may be good or vicious. In the former event the patient may be congratulated upon the issue of the arthritic trouble. Vicious positions are the straight in the case of the elbow, the rectangular in the hip and knee. In the case of the two latter there are often combined with the antero-posterior deflection considerable rotation, abduction, or adduction.

Pain in fibrous ankylosis is a very variable symptom. If the periarticular tissues have been implicated in the inflammatory process, any movement of the joint is painful. Considerable pain, swelling, and recurrence of the inflammation follow ill-advised attempts to break down unsuitable cases of ankylosis, and much discomfort is often caused by the application of extension instruments to the same class of cases. During the application of apparatus pain is often felt, both at those spots where the pressure of the instrument falls and in the joint itself. The extent of the deformity and disability can be readily estimated by the surgeon.

The prognosis can best be determined in the case of fibrous ankylosis by an examination under an anæsthetic. If with the employment of very slight force the adhesions readily give way, and but little heat, pain, or swelling follow, then a good result may be looked for when passive motion, douching, and massage are persevered with. But it is not the remotest use breaking down adhesions once and then sending the patient away. Passive motion must be employed within a day or two of the operation and steadily continued. Thus only can a successful result be obtained. If the ankylosis appear to require any powerful movements to overcome it, then all attempts should be at once desisted from, and the patient advised that nothing more can be done for him except it may be excision of the joint.

The *treatment* of fibrous ankylosis, excepting tuberculous, may be carried out in three ways: (1) Frequent and gentle daily passive movements, with massage and douching. (2) Rapid manipulation under an anæsthetic, succeeded by passive movements, massage and douching. Preliminary tenotomy and fasciotomy are often of service. The indications for this line of treatment have already been spoken of. For its ultimate success the full co-operation of the patient must be obtained, and he must be told that the passive movements will be painful, but must be steadily persevered in if the increased movements obtained after the anæsthetic are to be permanent. (3) Gradual extension by weights and instruments. From experience I do not think that any particular advantage in the direction of increased movement is obtained by these means. If the fibrous ankylosis is so firm as to require the force obtained by screws and racks to make it yield in some degree, then this treatment had better not be undertaken. Apart from the questions of time and expense, the results are often disappointing to the surgeon and positively detrimental to the patient.

In some instances fibrous ankylosis may be due to unwillingness on the part of the patient to move the joint after a slight attack of synovitis, or to want of firmness on the part of the medical attendant in insisting on the patient doing so. Particularly is this the case in some instances of severe spasm and in inflammation of a joint associated with fracture in the neighbourhood. Such cases drift about until they fall into the hands of the "bone setter," who, with one jerk, relieves the patient of his or her disability, and arrogates to himself the credit of "putting in a dislocated bone."

The directions in which force should be applied to the various joints may be briefly described as follows:—

**The Shoulder.**—The forearm is flexed on the arm, and the limb is kept as close as possible to the chest. The surgeon with his left hand fixes the scapula, and with the right hand firmly grasps the patient's elbow, or the forearm immediately below. The humerus is then rotated inwards and outwards until the head is felt to move freely in the glenoid cavity. The arm is then adducted and carefully abducted, and finally circumducted, with the limb as near the chest as possible. It is finally raised. All these movements should be carried out at one sitting. The anæsthetic must be repeated, and the former gain of movement secured and extended. Numerous accidents have occurred, owing to the employment of too much force, such as fracture of the neck of the humerus, rupture of the artery and vein, rupture of some of the cords of the brachial plenus and of muscles and tendons. After the anæsthetic I occasionally give an injection of morphia in neurotic people on account of the pain.

**The Elbow.**—With one hand, the left, fixing the affected elbow, the fingers pressing on the olecranon and the thumb on the head of the radius, the surgeon holds with the other hand the patient's wrist, and makes quick, short movements, first of flexion and then extension. If increased movement be secured, then pronation and supination are performed. It is well not to commence with extension, the danger being that the head of the radius may be displaced forwards.

**The Wrist.**—First flexion and then extension are tried in short quick movements, and finally circumduction.

**The Fingers.**—Each joint of a finger is flexed separately, and finally all the fingers together. At the metacarpo-phalangeal joint full flexion and extension are carried out.

**The Hip.**—The pelvis should be firmly fixed by an assistant, and short flexion movements attempted. If any success is obtained extension may be tried. In order to free the joint in the directions of abduction and adduction some of the tendons around the joint will need section. The majority of ankylosed hip-joints are tubercular, and one would hesitate to give any assurance of final success unless the history were perfectly clear as to the absence of that form of affection. If there is limited movement in the joint it is well to advise the patient not to submit himself to forcible manipulation, as in time additional flexibility of the lumbar spine will more than compensate for the loss of movement at the hip.

**The Knee.**—If the patella is fixed no good can come of forcible manipulation. The fibrous bands are too extensive. If it is not so and the joint is flexed, then the tendons and numerous fibrous bands at the back of the joint should be divided, preferably by the open method, and flexion movements tried, to be followed by extension. If the ankylosis be of long standing and the limb bent at right angles, the under surfaces of the condyles lengthen, and it is impossible to bring the tibia into a right line with the femur. In such cases the extremities of the condyles have been cut away. Various accidents have occurred in the attempt to straighten ankylosed knees. Among them are rupture of the popliteal artery and vein, tearing of the soft tissues of the ham, separation of the lower epiphysis of the femur and upper of the tibia, or rupture of the quadriceps tendon.

**The Ankle.**—If the joint is ankylosed near the right angle the movements in the tarsal and meta-tarsal joints will compensate largely for the deficiency at the ankle. If in extension, manipulation with section of the tendo-Achillis should first be attempted, and failing that excision of the astragalus or of the ankle-joint itself.

In bony ankylosis, when the position is bad, a linear or wedge-shaped osteotomy will often improve it. In the case of the shoulder, when the arm is abducted or adducted to a considerable degree, section of the humerus or excision of the joint will rectify it. But if the arm is by the side, it is questionable if it be not better to let matters remain in *statu quo*, as the movements of the scapula are so full and free. An elbow ankylosed in the straight position should be excised, and attempts made to secure movement by a false joint, or failing this, the parts should be allowed to ankylose at a right angle. Osteotomy, however, has secured, in the able hands of Mr. W. Adams, a great triumph in rectifying those distressing cases of rectangular bony ankylosis at the hip.

Adams' Operation of Section of the Neck of the Femur: By the surgeon whose name it bears this operation was first performed subcutaneously. Whether it be done in this way, or by a method more or less open, all antiseptic precautions must be observed. The details of the operation are as follows: The patient lying on the side opposite to the ankylosis, and the pelvis and limb being steadied by an assistant, the top of the great trochanter is felt for. About one inch above this an osteotomy knife is entered and carried downwards and inwards on the flat, until the anterior aspect of the neck is felt. The edge is then turned towards the bone, and cuts firmly on to it. Before withdrawing the knife the saw designed by Mr. Adams for the operation is passed along the knife, and the neck of the femur is then sawn through. If the neck has been accurately hit by the knife and saw the section of the bone should not occupy more than four to five minutes. Any tendons or bands of fascia which resist extension of the joint are then divided, and the limb is placed in a long Désault's splint. A very successful case was operated upon by me, in which I found it better to make an incision about one inch above the trochanter and to burrow with the finger down to the neck of the bone, guiding the saw into place. Mr. Adams' operation is by no means so easy to perform as might appear from the description given by its author. The neck of the femur is not easy to "hit" with the knife, and the tendency is to make the section at the junction of neck and great trochanter. In that event the time occupied in section varies from half to one hour, especially if the bone is sclerosed.

The operation is not suitable in children, in whom the neck of the femur is ill-developed, and, it may be, is partially destroyed by ulceration. The best results are obtained for ankylosis after rheumatic fever and acute suppurative arthritis.

Gant's Operation, or Infra-Trochanteric Osteotomy of the Femur: An osteotomy knife is passed to the outer part of the anterior aspect of the bone at the base of the great trochanter. The saw is entered along the knife, and the outer two-thirds of the femur divided. The remainder is then snapped by carrying the limb inwards.

When practicable it appears that Adams' operation is preferable to Gant's, especially when the deformity is rectangular or nearly so. Operating by Gant's method for a deformity of this severity is apt to leave a very ugly angle in the shaft of the bone, and may be followed by non-union.

For osseous ankylosis of the knee, a linear osteotomy in moderate cases, and removal of a wedge-shaped portion of bone in severer cases with division of the flexor tendons, will serve to remedy the deformity to a considerable extent.

